# How to analyse longitudinal data from multiple sources in qualitative health research: the pen portrait analytic technique

**DOI:** 10.1186/s12874-019-0810-0

**Published:** 2019-08-02

**Authors:** Laura Sheard, Claire Marsh

**Affiliations:** Bradford Institute for Health Research, Bradford Teaching Hospitals, Bradford, BD9 6RJ UK

**Keywords:** Qualitative, Longitudinal, Health research, Methodology

## Abstract

**Background:**

Longitudinal qualitative research is starting to be used in applied health research, having been popular in social research for several decades. There is potential for a large volume of complex data to be captured, over a span of months or years across several different methods. How to analyse this volume of data – with its inherent complexity - represents a problem for health researchers. There is a previous dearth of methodological literature which describes an appropriate analytic process which can be readily employed.

**Methods:**

We document a worked example of the Pen Portrait analytic process, using the qualitative dataset for which the process was originally developed.

**Results:**

Pen Portraits are recommended as a way in which longitudinal health research data can be concentrated into a focused account. The four stages of undertaking a pen portrait are: 1) understand and define what to focus on 2) design a basic structure 3) populate the content 4) interpretation. Instructive commentary and guidance is given throughout with consistent reference to the original study for which Pen Portraits were devised. The Pen Portrait analytic process was developed by the authors, borne out of a need to effectively integrate multiple qualitative methods collected over time. Pen Portraits are intended to be adaptable and flexible, in order to meet the differing analytic needs of qualitative longitudinal health studies.

**Conclusions:**

The Pen Portrait analytic process provides a useful framework to enable researchers to conduct a robust analysis of multiple sources of qualitative data collected over time.

## Background

Longitudinal qualitative research (LQR) is said to be that which focuses on experience over time, with change being the key focus of analysis [1]. Alongside understanding what change has happened, LQR explores how and why change happens within a socio-cultural context [2]. Practically, LQR can be understood as having two purposes: to collect data about a phenomenon over two or more time periods, or an analysis which involves comparisons of data across time periods [3]. LQR has a steeped history in the social science arena, for instance in well-known datasets such as the *Timescapes* series in the UK [4]. It is starting to be used in health research and health services research. Within health research, LQR most often takes the form of illuminating illness or recovery trajectories of patients in order to inform future health care priorities [5]. This most often takes the form of ‘serial interviews’ with the same cohort of patients over a given time period, about a specific disease or condition [1–3, 5–9]. The emphasis is on repeated contact with the same participants over time. Descriptions of this particular method are almost to the exclusion of other ways of collecting LQR data. Little methodological work has been published in relation to how LQR can be undertaken in relation to evaluations, intervention assessment or embedded as part of a randomized controlled trial in health research (although some authors working in the social policy space have explored elements of the above [10, 11]). Simultaneously, there is a dearth of literature which examines LQR in relation to applied health research [1, 2], as opposed to health research with patients.

The extensive volume of data which LQR can capture, alongside the inherent complexity resident in it, is said to be problematic. Narrative methods require specific attention to detail and therefore may be unsuitable for studies with large numbers of participants [8] or a large amount of data. Managing large quantities of qualitative data has the potential for the researcher to feel like they are ‘drowning in data’ [12] with the path to interpretation fraught with complexity [6]. Management and analysis of large volumes of temporal data is a key consideration of LQR researchers [4]. Correspondingly, there are few ‘off the shelf’ procedures for analysing LQR leading to researchers being unsure with what to do with their data [4]. This can lead to research teams having to design their own bespoke analytic methods to meet this need [5]. Particularly, the analysis of multi-dimensional data is a challenge which is not well described or reported in the literature [1]. By multi-dimensional, this could mean any study which seeks to involve more than one qualitative method in a longitudinal manner, for instance, multiple instances of interviews and ethnography over time. This challenge of reporting could be because this field is in its relative infancy, with LQR studies involving multiple methods in applied health research only starting to appear in the literature in the past few years [13–17].

It is useful to look at the current state of play with regard to LQR analysis. Authors working in the health LQR sphere who have published their analytic strategy tend to have, across all data sources, undertaken a thematic or constant comparison analysis [7, 15], a narrative approach [5, 6, 8] or deductively coded against an existing conceptual framework or taxonomy [14]. Explicit and careful attention was paid to the analytic process in a LQR study conducted in England about how motivational interviewing for depression after stroke may be effective [7]. ‘Parallel-serial memoing’ was the resultant technique developed and allowed a consensus to develop across different researchers in the same team. The focus was placed on how different researcher’s interpretations of the same dataset can be coherently brought together over time. The LQR dataset was based on one data source; transcripts of several motivational interviewing sessions. The research team conducted a thematic analysis based on the serial memos they developed in parallel to each other. A study in New Zealand conducted repeated interviews over 24 months with patients who had suffered a traumatic brain injury, and also their family members [5]. The research team describe using a narrative style analytic approach using “case sets” (one case set per participant) whereby transcripts at the 12 and 24 month time points were coded based on codes developed a priori from the six month time point transcripts in order to capture change or maintenance. The analysis underpinning a LQR study undertaken with first time parents in Austria is one of the few published accounts of how multiple and sometimes differing perspectives on the same topic over time can be analysed in a relatively systematic manner [18]. However, this articulated analysis relied on just one method – serial interviewing.

Despite the advances in the LQR analysis field described above, concrete descriptions of how research teams coherently and meaningfully *integrated* and made sense of the data *over time from different sources* are largely absent and elusive. Subsequently, there is minimal practical guidance given to researchers who may want to undertake this task. This risks the researcher approaching the analytic stage of a LQR project with a lack of described techniques in order to concentrate the data into a sufficiently meaningful *focused account*. This focused account could take many different forms. For example, it could portray how a team of healthcare ward staff interacted with an intervention over the period of an 18 month study, using data collected from in depth interviews and ethnographic field notes. Equally, it could pertain to how an individual GP utilizes a new software programme, based on think aloud interviews and non-participant observation over a 12 month implementation period. Critically, this focused account should aim to integrate data from all methods used in a LQR study in order to make sense of ‘what happened {to the GP or ward team} during the lifecourse of the study’ (changes over time) but also ‘what happened across the whole dataset at different time points’ (comparisons between GPs or ward teams at any specified point in the research process). It should aim to do this whilst maintaining a distilled version of the richness embodied in the data sources rather than a reductionist, dispersed account. It has been stated that analytic strategies which purport the first stage as coding or sorting text into discrete units of meaning risk stripping contextual richness away [19] and ‘breaking apart’ a participant’s story [6].

We have devised an analytic process which speaks to the above issue. It is called a *pen portrait* and has been used by the authors of this paper to successfully concentrate a large amount of longitudinal qualitative data into a focused account, in a previous empirical study [17]. The aim of this paper is to describe and explicate the process of creating and using pen portraits to conduct an analysis of LQR data.

## Methods

### Wider study method

In order to provide context for how and why we developed the pen portrait method for use in health research, an overview of the data collection which took place in the original, wider study is needed. We conducted a large randomised controlled trial involving 33 hospital wards across five hospital sites in the North of England, between 2013 and 2014. The trial tested whether a complex patient safety intervention led to improvements in key patient safety outcomes over a 12 month period. Wards were randomized to intervention or control group, with 17 in the former and 16 in the latter. In brief, the intervention gathered real time feedback from patients about their perceptions of safety on the ward, fed this data back to teams of ward staff via a structured report and then ward staff met in an action planning meeting (APM) and were tasked with making improvements to patient safety. They had the assistance and guidance of a facilitator during the APM. This process described above happened twice during the 12 months of the study, hence we were looking at change over time per individual ward team alongside comparing different ward teams at similar time points. The trial found no difference between intervention and control wards based on primary outcomes at 12 months [20].

An embedded qualitative process evaluation collected data between August 2013 and November 2014. The main a priori research question pertinent to the process evaluation was: “where does the intervention work, how and why?” Three main sources of qualitative data were analysed for this purpose. These were:

- Voice files of the taped APM discussion
- Facilitator’s field notes about the APM
- Semi structured telephone interviews six months after the APM.

APMs ranged in length from 27 to 80 min, with an average of 43 min. Our purpose in examining the APM voice files was to focus on which areas of patient feedback the ward staff made action plans about and which they chose not to. Facilitator’s field notes aimed to capture implicit dynamics about the APM that may not have been visible in an examination of the taped discussion. Telephone interviews had the core function of assessing whether action plans had been successfully implemented or not and the factors surrounding this. They were structured and usually short averaging around 15 min. The process evaluation methodology is described in significant further detail elsewhere [17]. Overall, we chose to focus on understanding how and in what ways the 17 ward teams engaged with the intervention over time. For our purposes, engagement with the intervention was classed as different to implementation of the intervention. The process evaluation findings can be consulted in detail elsewhere [17]. Briefly, we found that there was a general dilution of intervention implementation across the 17 intervention wards because ward teams engaged with the intervention in highly variable manners. This can be seen in the range of engagement typologies which arose from the qualitative dataset. Ultimately, faciliative processes put in place by the research team were potentially inadequate to enable successful engagement of ward teams with the intervention.

### Our analytic problem

We approached our analysis feeling somewhat overwhelmed by the volume of qualitative data in front of us, collected over the course of 15 months. We had of course been undertaking a tacit analytic process alongside data collection, which guided data collection as it went along. We did this by meeting regularly and discussing verbally the key issues that were arising from the data collection, deepening our implicit understanding of what the data was telling us over the duration of the study.

**Fig. 1:**
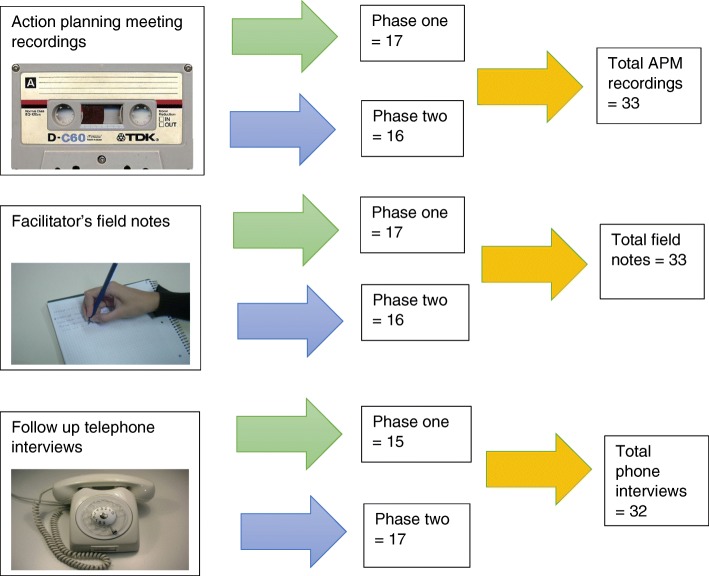
Our qualitative data sources (photos from Wikimedia Commons)

We came to an understanding that our ultimate problem was how to bring together the wealth of qualitative data that had been collected without losing richness. There were two cycles of feedback and action planning in the study so we had – mostly - two sets of recordings, field notes and telephone interviews for the 17 intervention wards (albeit one team who did not meet in an APM the latter phase and two teams who did not take part in a telephone interview in the former phase). That amounted to: 33 APM voice files, 33 sets of field notes and 32 telephone interviews. See Fig. 1 for a visual illustration of this.

In addition to volume, we also felt besieged by the complexity resident within the dataset as most wards waxed and waned regarding their engagement over time. The three different methods were designed to complement each other but in a minority of cases, the field notes written by the facilitator contradicted the tone or ethos of the recorded conversation during the APM. This was sometimes because the facilitator had picked up on unsaid subtleties in the interaction between ward staff and between staff and the facilitator during the course of the APM. How to deal with all the above issues across the dataset led to an analytic puzzle. At times, it seemed tempting to reduce our dataset down to one or two methods so that we could compare and contrast more easily across the wards and so the inherent background noise of volume and complexity would die down. However, we felt this would be an injustice to the rich data we had collected alongside being potentially unethical to our participants in gathering data from them which we then did not use. We looked to the literature to guide us because, as noted in the Background section, applied health research is becoming increasingly interested in using LQR approaches. We found nothing practical to assist us as to how to analytically integrate multiple qualitative methods collected over time. This led to us devising our own bespoke analytic process.

## Results

### Our solution – pen portraits

The primary purpose of a pen portrait is to document the journey, story or trajectory of the focus of enquiry in a more or less linear, narrative fashion over the life course of the study. The fundamental principles of this documentation process are to:

- draw on all of the methods used
- narrate interactions, impressions and events of importance which occur at key time points
- describe change occurring over time, as relevant
- a well-rounded, holistic account.

**Fig. 2:**
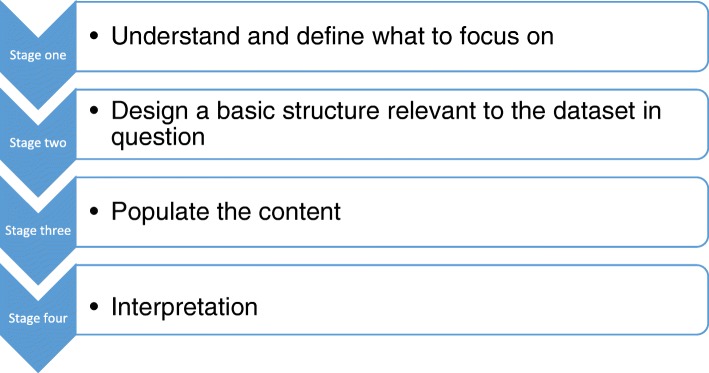
The four key stages of the pen portrait process

We intend the below stages as a guide only, open and welcome to modification, rather than a prescriptive diktat. We provide commentary from our own experience of developing the pen portrait method throughout the below stages in order to give context and assistance to the reader. The four key stages of the pen portrait process are detailed in Fig. 2.

#### Stage one – understand and define what to focus on

It is likely that by the time LQR data collection is fully underway that the researcher has intuitive ideas about the main themes that are shaping up, particularly after a period of time spent in the field. Likewise, it is probable in an applied health research environment that data is being collected to answer research questions pertinent to feasibility, acceptability, receptivity or engagement, alongside ‘what works, for whom, when and why?’ Therefore, understanding and defining the focus for analysis is likely to already have implicitly been undertaken but this should be formally explicated into a working document, which differing members of the research and analysis team can come to consensus over. It is essentially a process of de-mystifying what the main crux of the analytic endeavour will focus on so that the pen portrait serves as a useful resource rather than a narrative catch all, which may then become confusing rather than helpful later on in the process. Practically, a research team may need a series of meetings, with reflection and discussion in between times, to undertake this preparatory work. We include this first important stage as a result of trial and error on our part as, admittedly, we began our analytic process in a confusing manner as we were conflicted about what the core focus of analysis should be. We had been implicitly aware throughout the data collection process for the process evaluation that ward staff *engagement* with the intervention was a critical factor on a meta level across the dataset. But we came to this conclusion rather late having wasted time and effort going around in circles because we had not *explicitly defined* the core focus of our analysis upfront.

#### Stage two – design a basic structure relevant to the dataset in question

The structure for writing a pen portrait is important and time should be taken to develop something that works for all those who will be using it. The key idea behind the structure is simplicity, allowing the narrative account to become relatively free flowing and open without attempts to stifle or unnecessarily quantify the qualitative data. Think ‘dear diary’, rather than a rigid proforma or tick box exercise. The purpose of the pen portrait process is to allow for inductively generated findings to arise from multiple sources of data collected over time. Therefore, we would advise against devising a structure based on an existing conceptual framework or theory which is not an essential part of the core focus. This is because we believe this sort of deductive a priori structuring can stifle the inductive process. This is not to say that concepts and theory cannot be tacitly brought into the analytic process, far from it, but that we need to be clear that deductive coding against constituent parts of existing theory is not what we are trying to accomplish here.

**Fig. 3:**
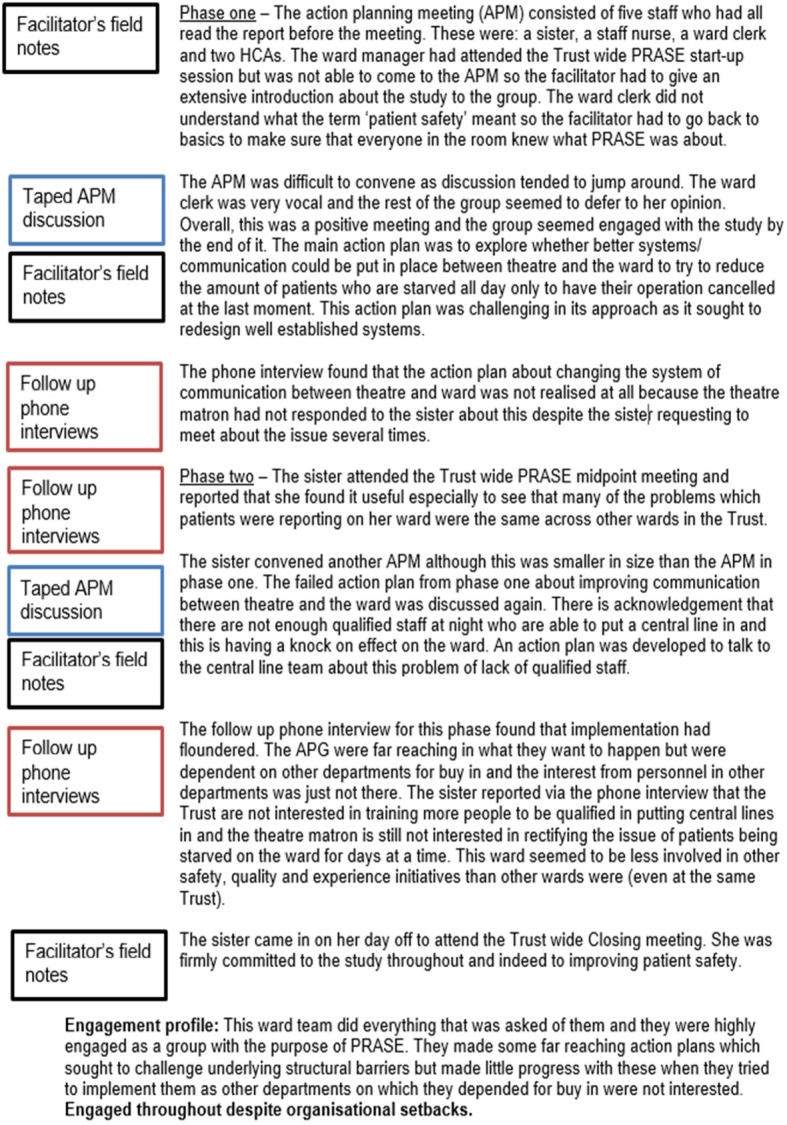
Holly ward pen portrait

Our structure devised for the patient safety study was extremely simple and worked for our purpose. We will now work through the most pertinent points of stage two with reference to the worked example in Fig. 3. The trial had two phases so we detailed material under a ‘phase one’ and then ‘phase two’ heading, with an ‘engagement profile’ at the end which sought to conclude each ward’s primary engagement style with the intervention. On later reflection, we would have probably added a section between phase one and phase two which documented our impressions of a key meeting which most ward staff attended. This is because we tended to write our impressions about this meeting at the start of phase two, which is slightly erroneous. Readers will be able to see in Fig. 3 that we did not force inclusion of all sources by having a prescriptive structure which made it compulsory for material to be included at each stage and from each data source. We deliberately chose not to do this as we felt it would unnecessarily fracture the narrative picture we were trying to build of each ward’s engagement trajectory if each data source was portrayed as disjointed accounts and not part of an overall story. For the purposes of demonstrating a worked example, in Fig. 3 we detail which individual source of data each part of the content was drawn from (annotated down the left hand side of the Pen Portrait for Holly ward). As the reader can see, sometimes content came simultaneously from two different data sources.

Pertaining to length, we tried to keep the whole account – including the summary - to less than two sides of paper, although most were shorter than this. It would be pertinent for research teams to pilot their pen portrait structure on a few test cases and then revise as appropriate. Colleagues within our department have started using our pen portrait methodology to assist their analysis of LQR datasets. Louch et al. (2018) [16] takes a slightly different approach to the structure by providing a longer summary per ward at the start of the document and then includes focused material on action planning (a key process in their study) and then a commentary on barriers and facilitators.

#### Stage three – populate the content

**Fig. 4:**
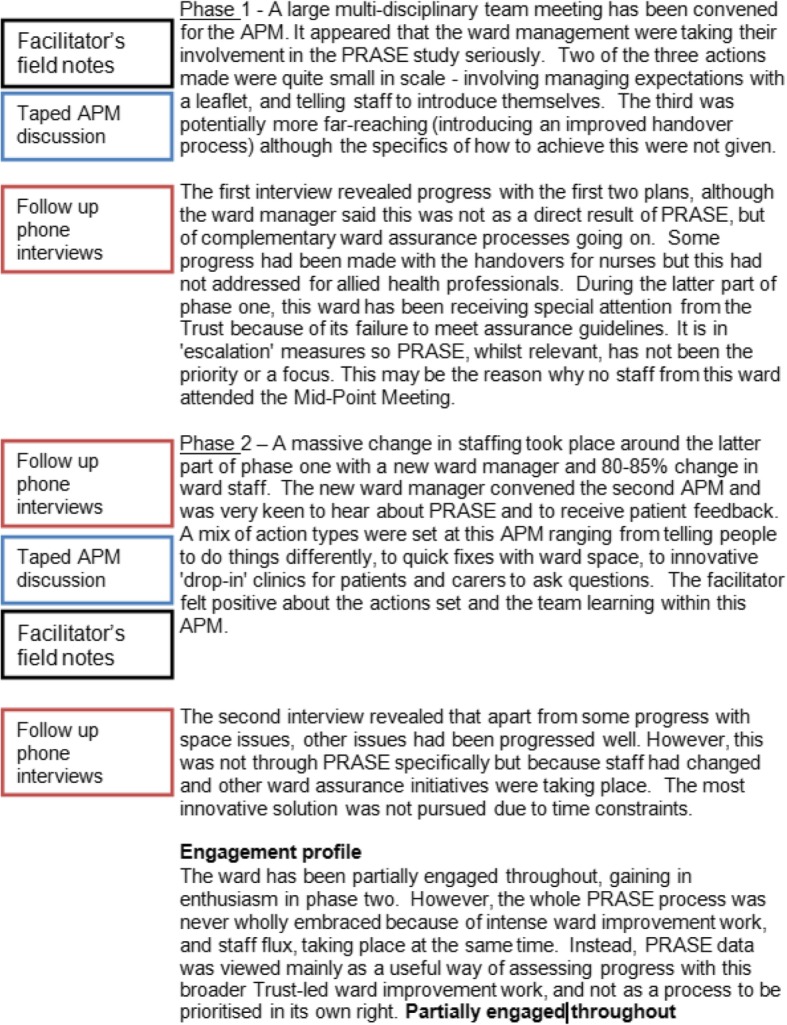
Chestnut ward pen portrait

The content of what to include in a pen portrait is highly individual and relative to the study matter at hand. In general, we would advise the content to start off descriptive but be discerning. Start to get a feel for what is superfluous or too minutiae-like and is detracting from the big picture. A fundamental part of the pen portrait approach is to try and draw on all of the methods used and this rationale is a key reason why we devised this analytic tool. Therefore, in our worked example, we looked across each of our three methods and pulled out the most salient points as related to our primary inquiry of engagement, one pen portrait per intervention ward involved in the process evaluation. We did this methodically in a step wise fashion by taking each phase of the study in turn. We started in a linear manner with phase one. First, we read over the research notes we had made based on the APM voice files and jotted down key impressions, interactions or progress as they related to engagement with the intervention. We went back to the original audio files, where necessary, to clarify particular aspects. Second, we paid attention to the facilitator’s field notes of the same meeting. Third, we looked at the researcher’s summary of the telephone interview where participants were asked about whether or not their action plans had been achieved, and the context surrounding this. Again, we revisited audio files as necessary. Additionally, we added small elements of tacit knowledge into the pen portrait but only if this served to enhance the narrative. An example of this would be a researcher’s interaction with one of the ward staff participants on a different day to when the APM took place, which may explain an underlying reason as to why a course of action was taken (or not) during the APM but was not specifically vocalised in the meeting itself. This would otherwise not have been captured in any of the three formal methods. Once we had considered material from all three methods and tacit knowledge (where relevant) in note form, we wrote this up into a summative narrative account striking a balance between description and making interpretive comment about engagement, based on fact. We then repeated the exercise for phase two. This part of pen portrait construction involved an element of creativity and the style was unique to each individual researcher working on the study, therefore difficult to distill. An example of another pen portrait from the same study is detailed in Fig. 4. It is interesting to note that Louch et al. (2018) [16] chose to use verbatim quotations when writing their pen portraits. This is an adaption to our original method as we did not include quotations in the original pen portraits.

An important element of this part of the process is to notice what is happening *between* different time points and to include commentary on this as the writing of the pen portrait proceeds. Change over time is a key part of LQR and if time points of the study end up being treated as separate chapters with little commentary on their linkage, then this is a lost opportunity. Once the process of writing a descriptive pen portrait has been completed, it is useful to then revisit earlier work to understand if elements of interpretation can be added in, relevant to the research question or focus. We usually added these straight into the main text but upon undertaking this process again, we would probably add them in as memos. It has to be said that drawing on all the methods used in a study is important but common sense must prevail. We would recommend that researchers do not laboriously include material for each and every method if the value and relevance of the data collected from certain methods clearly outweighs others. This will be for individual research teams to justify and reach consensus on.

After the pen portrait is considered complete, we found it useful to add a short summary section of no more than a few sentences. In our case, this was a very concise account of the ward’s engagement trajectory throughout the study. We did this due to our volume of pen portraits (as we had 17 intervention wards), so it served as useful aide memoire at a quick glance rather than having to read the entire document afresh and mentally ascribe a summary each time we returned to interrogate the data. Studies where the number of pen portraits is less may not feel they need to do this. Louch et al. (2018) [16] had seven wards in their study and chose not to write a formal summary section. This may be because they were more familiar with each ward given the relatively workable number of units of analysis.

#### Stage four – interpretation

A generic guide to interpretation is difficult to propose as this stage will depend heavily on the research questions of the topic in hand. The pen portrait methodology gives researchers the tool to manage potentially large volumes of complex LQR data into a narrative format which details the journey or trajectory of a chosen focus of inquiry throughout the length of a study. Therefore, the interpretation stage is largely similar to interpretation for many other qualitative studies. That is, researchers should be looking to go beyond the description (they now have a large amount of condensed, integrated description in front of them) and move towards developing conceptual ideas which offer explanations of what is occurring in the data. These conceptual ideas could be called ‘themes’ although it is important to note that, for our original intention, they tend to exist at a higher level than usual descriptive themes which are common in applied health research. Generation of themes and interpretation of data has been previously described in classic texts on this topic, such as Ritchie & Spencer (1994) [21] and Braun & Clark (2006) [22]. For an example of what is meant by a conceptual level analysis, see Sheard et al. (2018) [23].

The pen portraits can be used in diverse analytic manners. Each pen portrait can be taken in its entirety and compared or contrasted to the others arising from the same dataset. This allows for changes over time to be mapped across and between the units of inquiry. Calman (2013) [1] states that the analytic process should be focused on ‘processes and changes’ rather than snapshots. For our purpose, we were interested in the former and looked at the engagement trajectories of the 17 pen portraits and discovered the existence of five main engagement typologies regarding the ways in which ward teams engaged with key components of the intervention over time. These were: consistently engaged, partially engaged, increasing engagement over time, decreasing engagement over time and disengaged throughout [17]. The typology development entailed repeated reading of the 17 pen portraits to understand if their engagement could be viewed as strong or weak over time and whether this engagement type was static over time or had changed. LS and CM undertook an intense analysis session in order to categorise the 17 intervention wards into an engagement typology. Once we had developed these typologies, we used them as the basis for the rest of our analysis. Particularly relevant here is that we noticed that the engagement typology of ward teams at the same hospital was sporadic and not uniform, i.e. all wards were not either ‘consistently engaged’ throughout or ‘disengaged’ throughout. This led to an understanding that very senior management level support for the intervention had not necessarily filtered down to the level of the individual ward, particularly for wards at a hospital where senior management were extremely supportive of the intervention but the LQR data showed that some wards were not fully engaged.

## Discussion

In this paper, we have outlined the process of constructing a pen portrait with the intent that researchers may use this process in their own analyses of LQR data. We note four distinct stages: understanding and defining the core focus, designing the basic pen portrait structure, populating the content and, finally, interpretation. We give a large amount of instructive and - what we hope is helpful - detail in the first three stages but would encourage researchers to read more widely around issues of interpretation. Throughout our account, we provide pertinent examples of how we personally employed the stages described through reflections based on the dataset for which the pen portrait process was originally devised.

Braun and Clarke, in their 2006 classic text [22], state that a previous absence of clear and concise guidelines around thematic analysis may have led to an ‘anything goes’ critique of qualitative research. That is, by not discussing the ‘how to’ of analysis, techniques are therefore kept mysterious and elitist. Concrete advice on how to perform an analysis (of any kind) works towards making the analytic method accessible and democratic. We devised this bespoke analytic process because a search of the methodological literature provided no guidance whatsoever as to how an applied health researcher should go about the task of integrating large amounts of qualitative data from multiple sources over time, in a focused manner. LQR methods in the social sciences are seemingly well rehearsed [4] but their analytic strategies – where explicated and published - offer little assistance as they tend to focus on serial interviewing of the same participants over a period of years. In contrast, our project saw us collect qualitative data from 17 *teams* of people, using three distinct methods over an 18 month period. We needed an analytic method which was less about exploration and significantly more about answering specific research questions which were formulated a priori.

Several authors have noticed the above lack of instruction in the LQR methodological literature and have issued pleas for health LQR researchers to publish their methodological reflections in order to move the method forward [1, 2]. Calman et al. (2013) [1] have noted that the published literature relating to LQR is “limited in highlighting debates about LQR, focusing on the reporting on findings rather than developing debate about this emerging methodology”. We hope that by demonstrating the stages of the pen portrait method, and using a worked example to illustrate context, that we have answered this call and provided clear and concise guidelines. We believe that our specific contribution to moving LQR analysis forward is the novelty of proposing a technique which explicitly looks to integrate different methods over time. Some literature already exists with regard to researchers being able to make meaningful sense of change over time based on one method (such a serial interviewing of the same patients). Bringing data together from different qualitative methods, captured over time, is largely non-existent. This matters because applied health researchers are increasingly making us of multiple methods within the same study [13–17] but have no analytic instruction available to them. More important to us than bridging a gap in the methodological texts, our intention is that researchers are able to use the stages of the pen portrait as described in this paper practically, to develop a *focused understandin*g of what their LQR data is telling them.

Of great importance to us as developers of this technique, is the notion of adaptability and flexibility in its use going forward. To provide an analogy, we expect that we have given people the overall recipe for the dish but we expect that elements of the ingredients and their ratios will change over time, potentially leading to improvements in the flavour. We propose that the potential scope for the pen portrait technique is far-reaching and diverse. We see few restrictions on the ‘unit of analysis’ to which this could apply - in our case this was a ward, but it could equally be applied to an individual (following a health professional or a patient over time). In our case, we chose ‘engagement’ as our focus but we could have chosen other factors such as staff attitudes or perceptions. Outside of the realm of interventions, other foci could include patient experiences (e.g. disease symptoms or satisfaction). Finally, we believe the number of analytical units to also be flexible. In our case, we analysed the engagement trajectories of 17 intervention wards. We see no reason why the technique could not be applied to just one unit - e.g. one person or one ward - if the research question was not concerned with comparison between units but about a particular unit’s trajectory. A potential limitation is the number of units of analysis - and indeed the volume of data - that can be included which will be limited by the need for a largely consistent approach to the pen portrait steps. This issue may be hard to control in a very large study involving more than a small group of qualitative researchers.

We have already encountered a natural experiment in this regard as colleagues in our applied health research team have started to use the pen portrait technique, in the absence of any other structured manner of integrating multiple qualitative sources over time. Louch et al. (2018) [16] are the first to publish their findings (aside from our previous work for which the method was developed [17]). It is interesting to see how Louch et al. adapted our original premise by adding to and subtracting from elements of our approach which did not directly fit their analytic need. This demonstrates how the pen portrait technique has been taken forward as a *concept* rather than rigid proforma. Louch et al. go further than we did in developing distinct parts of the pen portrait which intuitively spoke to the niche needs of their analytic project. We hope others will adapt the technique for their own purposes.

## Conclusion

This paper presents Pen Portraits: a novel analytic process for qualitative data, collected from multiple sources and over time. We detail the four stages of how a researcher could use this technique and refer throughout to worked example, in order to provide context and guidance to the reader. In doing so, we believe a major gap in the qualitative longitudinal methodological literature has been addressed. We hope that by explicating the stages and detail pertaining to the development of a pen portrait that this analytic process can be taken forward and adapted by others to suit a variety of research purposes.

## Data Availability

Any requests for data should be directed to the corresponding author.

## Abbreviations

APM: Action planning meeting
LQR: Longitudinal qualitative research
